# Comparison and Optimization of hiPSC Forebrain Cortical Differentiation Protocols

**DOI:** 10.1371/journal.pone.0105807

**Published:** 2014-08-28

**Authors:** Christina R. Muratore, Priya Srikanth, Dana G. Callahan, Tracy L. Young-Pearse

**Affiliations:** 1 Center for Neurologic Diseases, Brigham and Women’s Hospital and Harvard Medical School, Boston, Massachusetts, United States of America; 2 Program in Neuroscience, Graduate School of Arts and Sciences, Harvard University, Cambridge, Massachusetts, United States of America; 3 Harvard-MIT Division of Health Sciences and Technology, Cambridge, Massachusetts, United States of America; University Of Melbourne, Australia

## Abstract

Several protocols have been developed for human induced pluripotent stem cell neuronal differentiation. We compare several methods for forebrain cortical neuronal differentiation by assessing cell morphology, immunostaining and gene expression. We evaluate embryoid aggregate vs. monolayer with dual SMAD inhibition differentiation protocols, manual vs. AggreWell aggregate formation, plating substrates, neural progenitor cell (NPC) isolation methods, NPC maintenance and expansion, and astrocyte co-culture. The embryoid aggregate protocol, using a Matrigel substrate, consistently generates a high yield and purity of neurons. NPC isolation by manual selection, enzymatic rosette selection, or FACS all are efficient, but exhibit some differences in resulting cell populations. Expansion of NPCs as neural aggregates yields higher cell purity than expansion in a monolayer. Finally, co-culture of iPSC-derived neurons with astrocytes increases neuronal maturity by day 40. This study directly compares commonly employed methods for neuronal differentiation of iPSCs, and can be used as a resource for choosing between various differentiation protocols.

## Introduction

Since the advent of human induced pluripotent stem cell (hiPSC) technology, numerous studies have utilized these cells for neuronal differentiation. Several groups have independently developed hiPSC neuronal differentiation protocols, often adapted from existing protocols for human embryonic stem cells (ESCs) or mouse iPSCs/ESCs [1]–[10]. These protocols are constantly being improved and revised, creating a plethora of techniques to differentiate hiPSCs to neuronal fates. The ability to differentiate, culture, and manipulate human neurons is of tremendous interest to labs seeking to study human neurodevelopment and neurological diseases. For a group that is new to stem cell culture and differentiation, the multitude of available neuronal differentiation protocols can be overwhelming. Here, we aim to directly compare some of the most commonly used techniques in human neuronal differentiation, using gene expression, cell morphology, and immunostaining to benchmark efficiency. We hope this study may provide useful information to aid in other groups’ future decisions regarding iPSC differentiation methods and reagents.

Many groups have taken advantage of somatic cell reprogramming technology to generate patient-specific iPSC lines in order to model neurodegenerative and neurodevelopmental disorders (reviewed in [11]). Furthermore, there have been many advancements in protocols to create neurons of a particular identity (e.g. motor neurons, dopaminergic neurons or interneurons) [12]–[18]. There are often multiple protocols to differentiate stem cells to a particular neuronal fate of interest. While a comparison of neuronal patterning protocols would certainly be informative, it is outside the scope of this study. Here, we focus on methods for differentiating iPSCs to a “default” forebrain cortical neuronal fate.

For the differentiation of iPSCs to forebrain neurons, two base protocols are often utilized: an embryoid aggregate-based technique and a monolayer dual SMAD inhibition method [8], [19]. In the embryoid aggregate procedure, iPSC colonies in iPSC media are allowed to form aggregates in suspension in the absence of exogenous growth factors or small molecules. The media is then changed at day 5 to a neural induction media with a DMEM/F12 base, containing non-essential amino acids, heparin, and N2 supplement, which supplies transferrin and insulin, among other components (“*N2 neural induction media*”). The primitive neuroectodermal aggregates are plated at day 7 onto an adherence-promoting substrate, and cultured for 10 days, promoting formation of definitive neuroectoderm. At day 17, neural progenitor cells, organized into neural “rosette” structures, are selectively removed from the plate and cultured in suspension. These neural aggregates are cultured in a similar neural induction medium, but with the addition of B27 supplement (containing biotin, DL alpha tocopherol, vitamin A, BSA, catalase, insulin, transferrin, and superoxide dismutase, among other components), cyclic AMP (cAMP), and insulin growth factor-1 (IGF-1) (“*N2/B27 neural induction media*”). After being cultured in suspension for 7 days, the neural aggregates are plated on an adherent substrate in a differentiation-promoting media. This “*neural differentiation media*” is made with a neurobasal base media supplemented with non-essential amino acids, N2, B27, cAMP, IGF-1, brain-derived neurotrophic factor (BDNF), and glial cell-derived neurotrophic factor (GDNF). Differentiated neurons are visible from day 25 onwards, and can be cultured as long as is desired for experimental purposes [19]. There exist multiple variations on this protocol, including aggregate formation techniques, the use of different plating substrates, neural progenitor cell isolation methods, and co-culture of neuronal cells with astrocytes.

The monolayer dual SMAD inhibition protocol [8] involves dissociating iPSCs and plating them as a feeder-free adherent monolayer before rapidly inducing neuroectoderm formation by antagonizing the bone-morphogenetic protein (BMP) and transforming growth factor beta (TGF-beta) signaling pathways (e.g. by using Noggin and SB431542, respectively). The morphogen Noggin and small molecule SB431542 induce conversion of hiPSCs or hESCs to a neural progenitor cell fate by day 7, in a neural induction media made with a DMEM/F12 base and insulin, N2, and B27 (“*3N neural induction media*”) [10]. At day 11, cells are dissociated and re-plated in neural differentiation-promoting media (“neural differentiation media,” defined above). Thus the media used by the dual SMAD inhibition protocol is largely similar to those utilized in the embryoid aggregate method. Two main differences exist between these two protocols: 1) morphogens/small molecules block the BMP and TGF-beta pathways in the dual SMAD inhibition protocol, and 2) the monolayer (dual SMAD inhibition protocol) versus the three-dimensional aggregate (embryoid aggregate technique) culture. The resulting timelines of these methods are also distinct, with neuroectoderm at day 17 vs. day 7, and neurons at day 25 vs. day 12 in the embryoid aggregate vs. dual SMAD inhibition protocols, respectively.

Multiple studies have utilized each of these methods, often with modifications, to generate human iPSC-derived neurons. These variations involve the use of different reagents at multiple stages of differentiation to achieve a common goal: culture of human neurons. It is not always clear from a published study why a particular method was chosen and how the method employed compares to other available protocols. Here we examine both the embryoid aggregate and dual SMAD inhibition protocols and compare commonly used experimental paradigms for aggregate formation, plating substrates, NPC isolation and expansion, and neuronal maturation. We evaluate these various techniques through the use of common metrics such as morphology, immunostaining and gene expression.

## Results

### Differentiation of Human iPSCs Into Neurons Using an Aggregate Method

**Figure 1:**
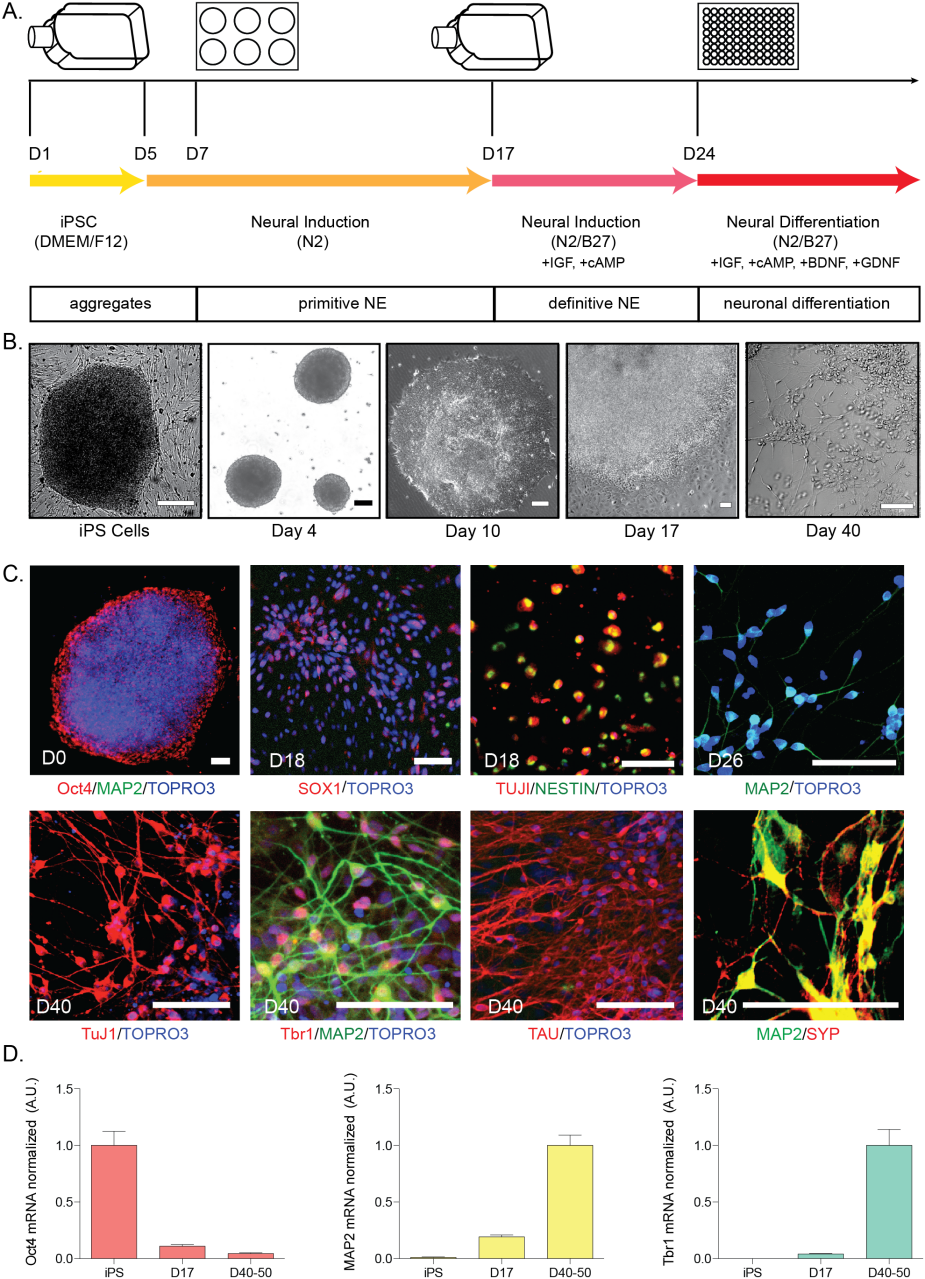
Embryoid Body Differentiation of hiPSCs. A) Time course of differentiation for embryoid aggregates. iPSC colonies were dissociated from mouse embryonic fibroblasts at day 1 (D1) and cultured as aggregates in suspension. Aggregates were plated onto culture dishes at day 7 (D7), forming primitive neuroepithelial (NE) structures. By day 17 (D17), definitive NE structures were present; NE structures were manually isolated and further cultured in suspension. Cells were plated for final differentiation at day 24 (D24). Arrows indicate media changes across differentiation. Boxes indicate differentiation state. This protocol was performed in 11 independent lines, with all lines performing similarly; representative images are shown. B) Bright-field microscopy images showing morphological changes spanning differentiation from the earliest time-point (iPSCs) to day 40 (D40) neurons. Scale bars from left to right: 100, 200, 200, 500, 500 µm. C) Cells were immunostained at various time-points during neuronal differentiation. Confocal microscopy images at days 0 (iPS colony), 18, 26, and 40. Scale bars = 100 µm. TOPRO3, nuclear marker. D) qPCR analysis of markers over differentiation. Ct data normalized to *GAPDH*. For Oct4: iPS n = 14, D17 n = 23, D40–50 n = 19 with data points all normalized to iPS; MAP2: iPS n = 15, D17 n = 25, D40–50 n = 26 with data points all normalized to D40–50; Tbr1: iPS n = 14, D17 n = 25, D40–50 n = 26 with data points all normalized to D40–50, from 6 independent differentiations. Data are represented as mean ± SEM.

To examine various differentiation strategies, we first utilized an embryoid aggregate protocol [19] originally based on methods developed for hESCs [20]. Fig. 1A shows the timeline schematic for the protocol, in which human iPSCs are differentiated to neuronal fates over the course of ∼40 days. Aggregates were formed by dissociating iPSCs as large clusters at day 1, followed by suspension in culture for five days in serum-free iPS media (without FGF2). At day 5, aggregate media was changed to N2 neural induction media. Aggregates then were plated on Matrigel for the formation of primitive neuroepithelial cells (Fig. 1B, day 10) in N2 neural induction media. At day 17, neural rosette structures were manually selected from plates and suspended in flasks for another week in N2/B27 neural induction media. This step aims to select for definitive neuroepithelial cells since many non-neuroepithelia cell types adhere to the flask. At day 24, aggregates were plated on Matrigel and allowed to mature for an additional 15–30 days in neural differentiation media.

In order to qualitatively assess the progression of differentiation, we performed immunostaining for various markers indicative of the differentiation process (Fig. 1C). Undifferentiated iPSC colonies expressed the pluripotent marker Oct4 (POU5F1), but lacked expression of neuronal cytoskeletal markers such as MAP2. The intermediate time-point day 18 shows the expression of neural progenitor markers Sox1 and Nestin. Neurons differentiated for 40 days express neuronal proteins such as MAP2, TuJ1, and Tau, the cortical marker Tbr1, and synaptic markers such as synaptophysin (SYP) (Fig. 1C, bottom row). Functional analyses were performed using a microelectrode array platform. Spontaneous potentials were observed at around 50 days of differentiation, as previously reported using this protocol [21]. In order to quantitatively assess and compare differentiation progression across multiple wells, qPCR was performed for multiple cell-fate markers (Fig. 1D). Data show that with an increase in differentiation time, mRNA expression of *Oct4* (*POU5F1*) decreases, while neuronal markers such as *MAP2* and *Tbr1* increase, and this expression pattern is consistent between wells of the same experiment and between differentiation rounds. To complement the qPCR data and determine the absolute percentage of neuronal cells derived using this method, the percentage of cells expressing MAP2 was quantified from immunostained wells, with 93% (±1.5 SEM) of cells expressing MAP2 by day 40.

### Generation of Neurons Utilizing Dual SMAD Inhibition in Monolayer Culture

**Figure 2:**
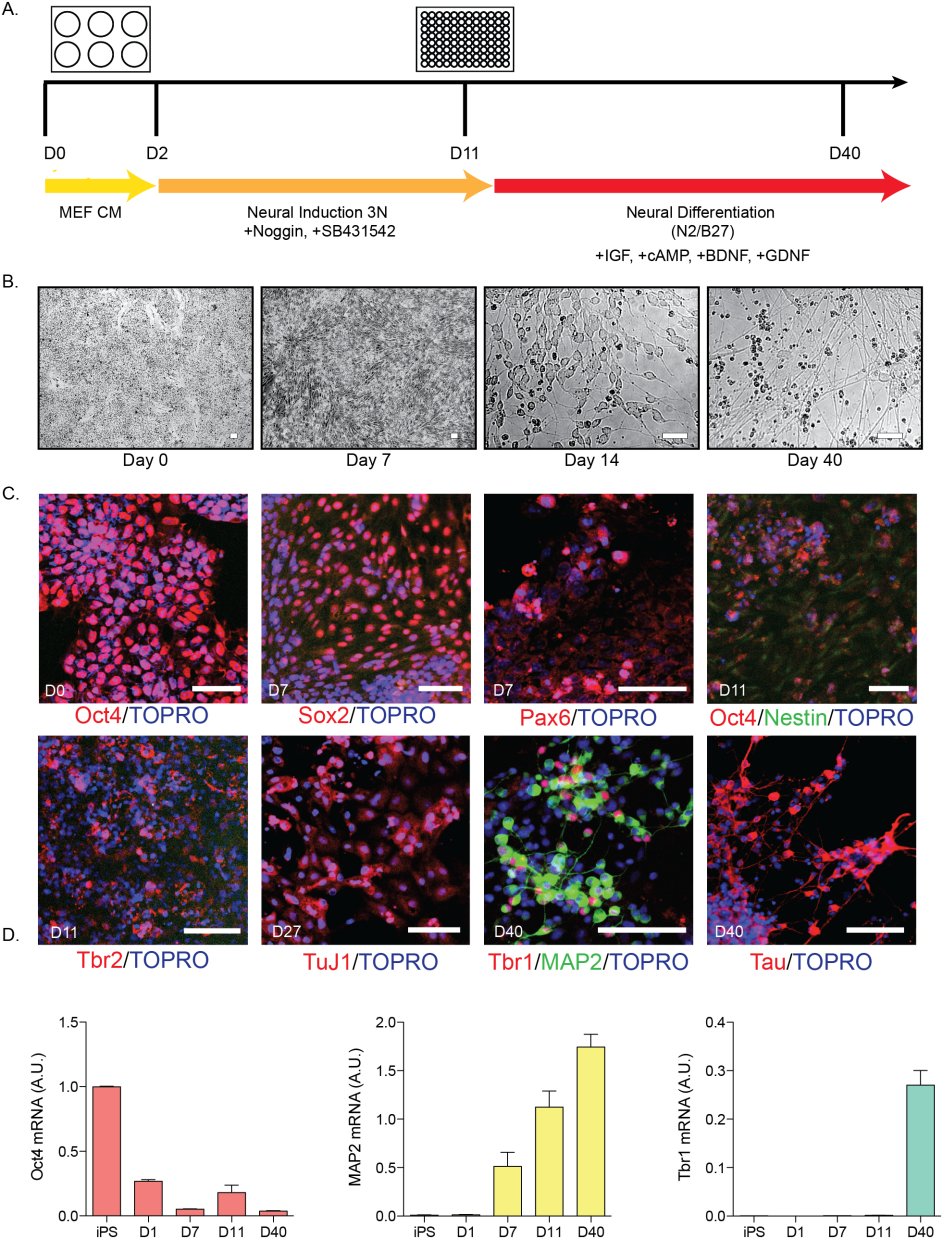
Monolayer Differentiation of hiPSCs. A) Time course of differentiation using dual-SMAD inhibition. iPSC colonies were dissociated from mouse embryonic fibroblasts at day 1 (D1) and plated as a monolayer. Small molecules and growth factors were added as indicated. This protocol was performed in at least 6 independent lines; representative images from the most efficient differentiations are shown. B) Bright-field images spanning differentiation from the earliest time-point day 0 (D0) to day 40 (D40). Scale bars = 50 µm. C) Cells were immunostained at various time-points during neuronal differentiation. Confocal images at days 0, 7, 11, 27 and 40. Scale bars = 100 µm. TOPRO, nuclear marker. D) qPCR analysis of markers over differentiation. Data normalized to *GAPDH*. For Oct 4: iPS n = 3, D1 n = 3, D7 n = 3, D11 n = 6, D40 n = 5; MAP2: iPS n = 3, D1 n = 4, D7 n = 4, D11 n = 6, D40 n = 5; Tbr1: n = 2, D1 n = 3, D7 n = 3, D11 n = 5, D40 n = 5. Data are represented as mean ± SEM.

We next sought to compare a monolayer-based protocol to this aggregate method. Fig. 2A illustrates the timeline schematic that was utilized, based on the technique of dual SMAD inhibition [8]. At the start of differentiation (day 0), iPSCs were dissociated to single cells and re-plated as a monolayer with a concentration of 20,000 cells/cm^2^ in MEF conditioned media, supplemented with FGF2. After cells reached 90% confluency, media was changed to 3N neural induction media supplemented with Noggin (200 ng/mL) and SB431542 (10 µM) [10]. Cells were split at day 11 using dispase and re-plated in neural differentiation media onto 96-well plates coated with Matrigel. The bright-field images in Fig. 2B illustrate the morphological changes over the course of differentiation. At day 7, the cells begin to form early rosette structures. After re-plating the cells at day 11, small processes begin to emerge (day 14), followed by more mature neuronal morphology at day 40 (Fig. 2B, last panel).

Both immunostaining and qPCR were employed to examine differentiation efficiency over time. Cells begin to express progenitor markers Sox2 and Pax6 at day 7 and Nestin and Tbr2 at day 11. From its maximal expression at day 0, Oct4 expression is markedly decreased at day 11 (Fig. 2C). From day 27 through day 40, neuronal markers Tau, MAP2, Tbr1 and TuJ1 are expressed. Based on quantification of immunostaining, approximately 45% (±4.6 SEM) of cells expressed MAP2. Similarly to the aggregate method, when we probed mRNA from harvested cells, *Oct4* (*POU5F1*) decreased over differentiation time while *MAP2* and *Tbr1* increased up to day 40 (Fig. 2D). However, this method often resulted in “failed” differentiations due to high levels of cell death between days 10–17 of differentiation. Neuronal differentiation using the dual-SMAD inhibition protocol without splitting led to cultures that either died or did not produce MAP2+ neurons (10/10 differentiation rounds), due to over-confluent cultures between days 10–17. However with a revision in the protocol that included splitting the cultures at day 11 (Chambers and Studer, personal communication), we observed MAP2+ cells in 3/5 differentiation rounds. Based on these initial results, we chose to focus upon optimizing the embryoid aggregate differentiation protocol.

### Comparison of Embryoid Aggregate Formation: Manual versus AggreWell

**Figure 3:**
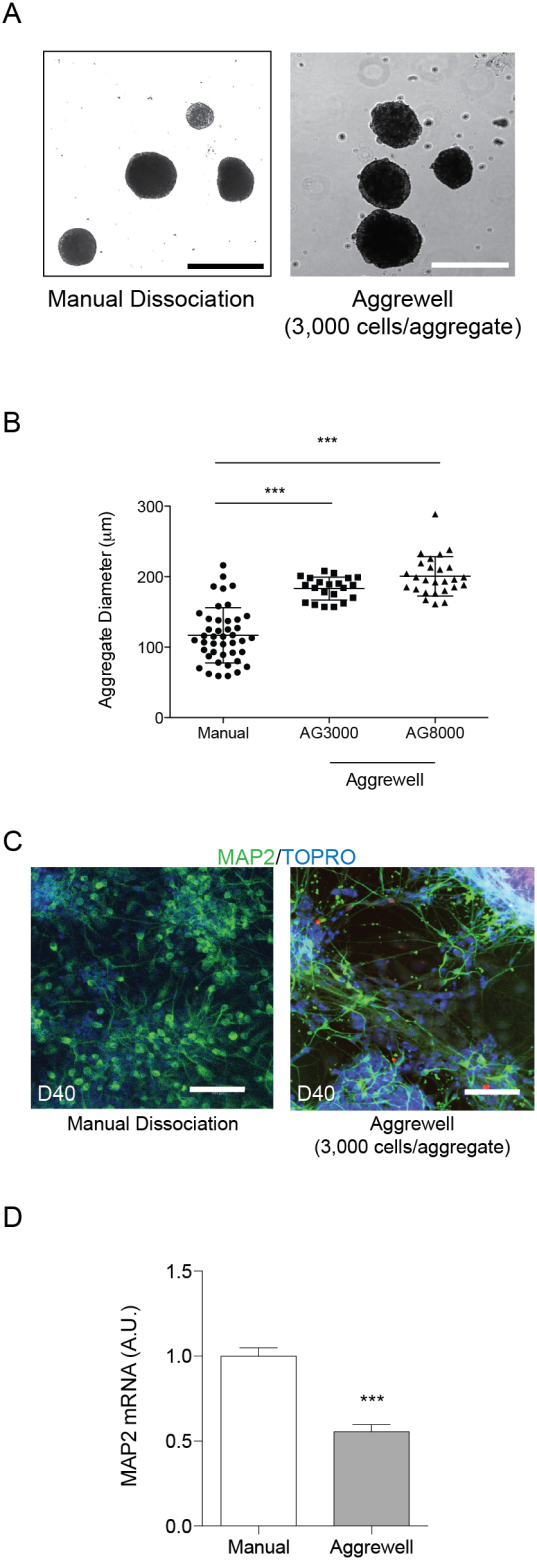
Comparison of Embryoid Body Formation. A, B) Embryoid bodies were either formed by dissociating iPSCs (using dispase and trituration) or by AggreWell plate technology, followed by culture in non-adherent flasks. B) Quantification of aggregate size from manually-formed or 3,000- or 8,000-cell aggregates. Mean diameter for manually formed aggregates = 118.3 µm; mean diameter for 3,000 cells/aggregate = 183.1 µm; mean diameter for 8,000 cells/aggregate = 195.2 µm. Scale bars = 200 µm. Data are represented as mean ± SEM, from 4 independent differentiations, n = 21–43. Significance determined by one-way ANOVA with a Tukey’s post-test: ***, p<0.0001. F-tests between groups showed significantly different variances, with p<0.05 between manual vs. 3,000 cells/aggregate and manual vs. 8,000 cells/aggregate. C) Immunostaining of day 40 (D40) neurons, following differentiation using either manual formation or AggreWell plates. TOPRO, nuclear marker. Scale bars = 100 µm. Representative images are shown. D) qPCR was performed using RNA harvested from day 40 cultures. Data normalized to *GAPDH* expression. Manual n = 10, AggreWell n = 10. Data are represented as mean ± SEM. Significance was determined by student's t-test: ***, p<0.0001.

We hypothesized that differentiation efficiency could be improved by creating embryoid aggregates of a more uniform size, using AggreWell plates. At day 0, iPSCs were dissociated manually using dispase and either resuspended in flasks or triturated and plated in AggreWell plates. With AggreWell plates, cells were force-pelleted into microwells by centrifugation. After 24 hours, dissociated cells formed aggregate structures and were further cultured following the protocol outlined in Fig. 1A. We made aggregates of two different types: 3,000 and 8,000 cells/aggregate. Manually formed aggregates consisted of varying shapes and sizes (Fig. 3A), whereas aggregates formed using AggreWell were visually more uniform in size and shape. These size differences were quantified by measuring the diameter of aggregates (Fig. 3B). The mean diameter for manually formed aggregates was 118.3 µm (±6.0 SEM), whereas the mean diameter was 183.1 µm (±3.6 SEM) for 3,000 cells/aggregate and 195.2 µm (±5.5 SEM) for 8,000 cells/aggregate. Both sizes of AggreWell aggregates were significantly larger than manually formed aggregates, and although there was a trend for an increased aggregate diameter between 3,000 and 8,000 cells/aggregate, it did not reach statistical significance. As the AggreWell system is designed to incorporate 3,000 versus 8,000 cells into each aggregate, the insignificant difference in aggregate size may reflect a difference in aggregate density, with 8,000 cells/aggregate being more densely packed than 3,000 cells/aggregate. Not surprisingly, the variance of aggregate size distribution was significantly greater with manual aggregate formation than with either AggreWell size. Immunostaining for MAP2 in cells following aggregate formation with the use of AggreWell is shown in Fig. 3C (right). Immunostaining at day 40 showed that approximately 46% (±1.6 SEM) of AggreWell-differentiated cells were MAP2+, compared to 93% MAP2+ cells with manually formed aggregate differentiation. Quantification of *MAP2* mRNA from day 40 neurons that were cultured in the AggreWell format also showed a corresponding significant decrease in *MAP2* mRNA levels (Fig. 3D).

### Comparison of Plating Substrates: Matrigel vs. Poly-o-laminin

**Figure 4:**
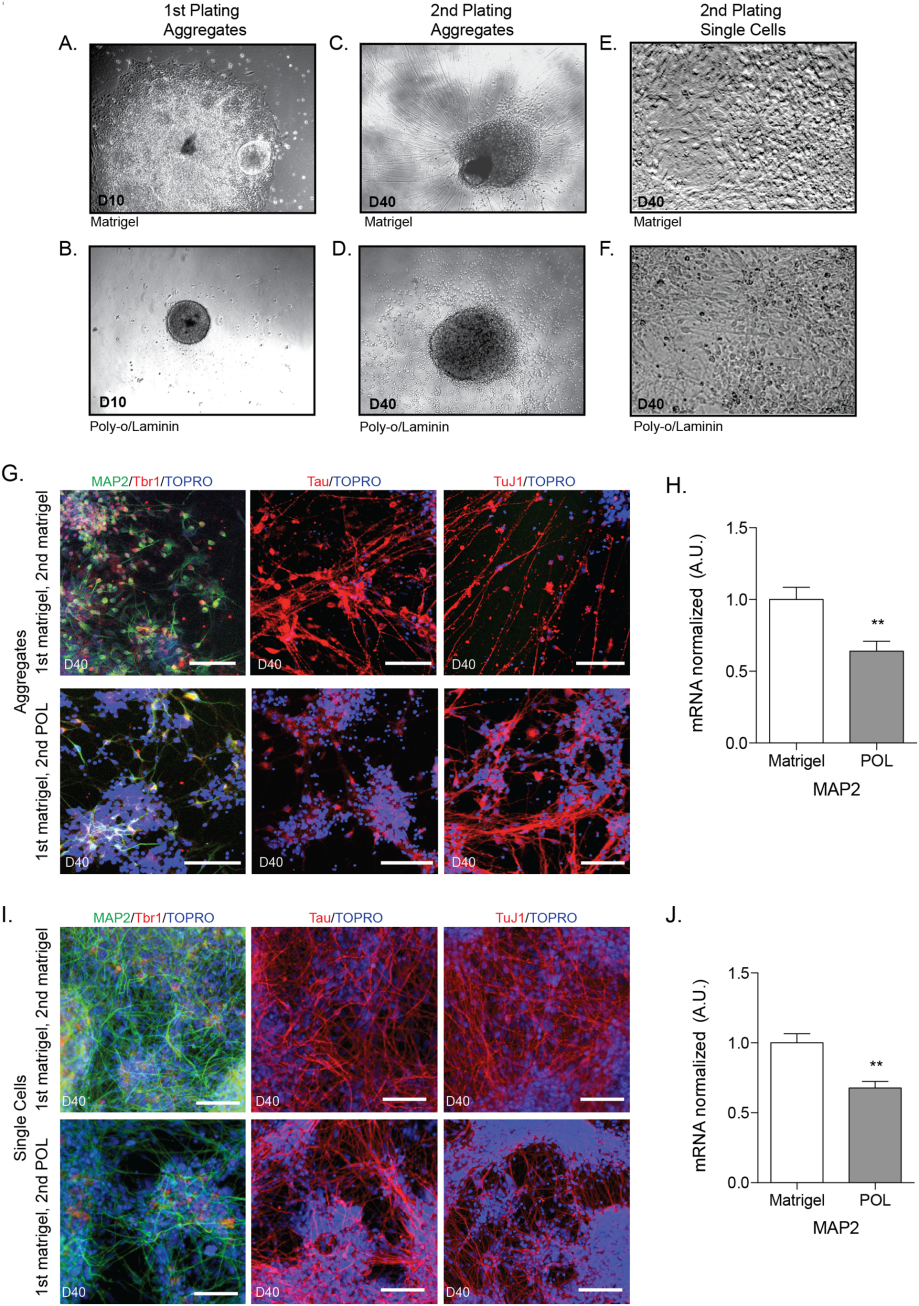
Comparison of Plating Substrates. Aggregates were plated on either Matrigel or poly-o/laminin (POL) coated plates at days 7 or 24. A, B) Aggregates plated at day 7 (D7) and imaged at day 10 (D10) on Matrigel (A) formed typical neuroepithelial structures, while aggregates plates on POL (B) failed to adhere after two days. C, D) Aggregates were plated on either Matrigel or POL coated plates for final differentiation on day 24 (D24) and imaged at day 40 (D40). Aggregates plated on Matrigel (C) exhibited an increased density of processes, while aggregates plates on POL (D) displayed increased cell body migration from the plated aggregate. E, F) Neural aggregates were dissociated at day 24 and plated on either Matrigel (E) or POL (F). G) Aggregates were plated on either Matrigel (top row) or POL (bottom row) at day 24 and allowed to mature until day 40, followed by immunostaining and confocal microscopy for neuronal markers. Scale bars = 100 µm. Representative images are shown. H) qPCR was performed using RNA harvested from day 40 cultures. Data normalized to *GAPDH* expression. Matrigel n = 10, POL n = 10. I) Aggregates were single-cell dissociated and plated on either Matrigel (top row) or POL (bottom row) at day 24 and allowed to mature until day 40, followed by immunostaining and confocal microscopy for neuronal markers. Scale bars = 100 µm. Representative images are shown. J) qPCR was performed using RNA harvested from day 40 cultures. Data normalized to *GAPDH* expression. Matrigel n = 22, POL n = 22. For H and I, significance determined by student’s t-test: **, p<0.01; ***, p<0.001. Data are represented as mean ± SEM.

The choice of plating substrates for differentiation varies among labs and protocols. By far, the two most commonly used substrates are Matrigel and a poly-ornithine/laminin combination (POL). We sought to compare the results of using Matrigel versus POL substrate at the two plating steps of the embryoid aggregate technique (Fig. 1A). We found that using Matrigel for the first aggregate plating (day 7) was sufficient to direct differentiation to a neuroepithelial fate (Fig. 4A). However, when we attempted to plate aggregates on POL at the same time-point, aggregates did not reliably adhere to the plate (Fig. 4B). For the second plating of neural aggregates at day 24, cells were plated on either Matrigel (Fig. 4C) or POL (Fig. 4D). For both plating substrates, aggregates were able to adhere to the plate, and cells with neuronal morphology were visible. However, aggregates plated on POL displayed sparser distribution of cell processes and more migration of cell bodies away from aggregates (Fig. 4D) compared to aggregates plated on Matrigel (Fig. 4C).

While plating aggregates for final differentiation induces efficient neuron generation, for some purposes it may be desirable to have a culture that is more monolayer in nature. For example, aggregates can interfere with imaging as it is difficult to visualize cells in or near large aggregates. In an effort to create a monolayer cell culture, aggregates were dissociated with accutase at day 24 and plated on either Matrigel or POL (Fig. 4E, F).

Immunostaining of day 40 differentiated neurons (aggregates) showed decreased MAP2 staining (Fig. 4G, bottom row) as well as low levels of Tau staining, in POL versus Matrigel-plated neurons (Fig. 4G, top row). TuJ1 staining appeared to be consistent between the two plating conditions. *MAP2* mRNA levels from day 40 differentiated neurons, plated on either Matrigel or POL, were quantified using qPCR (Fig. 4H). Cells from POL-plated aggregates expressed significantly less *MAP2* mRNA than cells plated on Matrigel. Based on immunostaining, approximately 56% (±3.5 SEM) of differentiated neurons expressed MAP2 on POL, compared to 93% MAP2+ cells with Matrigel plating.

Immunostaining of dissociated aggregates (single cells) revealed similar results to those seen in Fig. 4G. Dissociated single cells plated on POL exhibited less dense cultures than neurons plated on Matrigel, with less overall staining of neuronal processes (MAP2, Tau, TuJ1) (Fig. 4I, bottom row). *MAP2* mRNA from day 40 dissociated/single-cell neurons, plated on either Matrigel or POL, was quantified using qPCR. Dissociated cells plated on POL had significantly lower *MAP2* mRNA expression than cells plated on Matrigel (Fig. 4J).

### Comparison of Neural Progenitor Cell (NPC) Isolation by Multiple Methods

**Figure 5:**
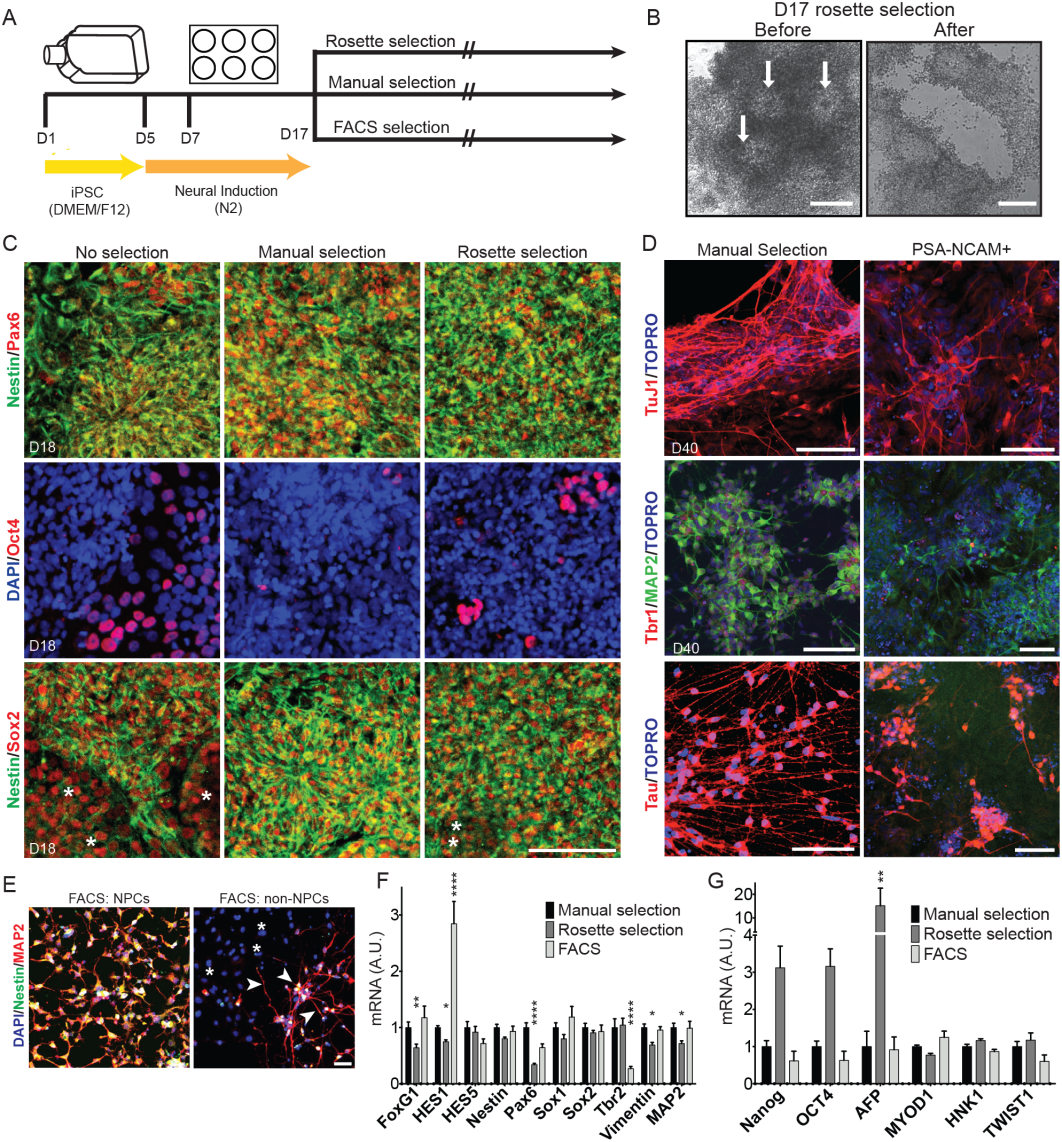
Comparison of NPC isolation methods. A) Schematic indicating the time course of differentiation and the techniques used to isolate neural progenitor cells (NPCs). Human iPSCs were differentiated for 17 days. NPCs were isolated by manual scraping of non-NPCs under a microscope (manual selection), using a proprietary neural rosette selection reagent (rosette selection), or by FACS for CD184+/CD44−/CD271−/CD24+ cells (FACS). B) Representative bright field images are shown for selection of rosettes using rosette selection reagent. White arrows indicate rosette structures to be isolated. After use of the reagent, rosettes are isolated. Scale bars = 100 µm. C) Immunostaining for various cell fate markers at day 18 after isolation at day 17. Asterisks in the bottom panel show Sox2+Nestin− cells. Scale bars = 100 µm. D) Day 17 NPCs were either manually selected or dissociated using accutase and processed for cell sorting. Manually selected or PSANCAM+ cells were plated on Matrigel for 23 days in neural differentiation media and immunostained at day 40 for neuronal markers. Scale bars = 100 µm. E) Day 17 cells were dissociated and subjected to FACS. CD184+/CD44−/CD271−/CD24+ cells (“NPCs”) and all other cells (“non-NPCs”) were plated on Matrigel and maintained in neural progenitor media for 20 days prior to immunostaining. Scale bar = 50 µm. F, G) RNA was harvested from cells at day 17 after isolation and used in the NanoString assay. Expression profiles of selected NPC fate markers (F) or other cell fate markers (G) are shown. Gene expression was normalized to the geometric mean of seven housekeeping genes. Data are represented as mean ± SEM. Data are from 5–6 independent differentiations and 3 lines, n = 6–30. Significance is shown compared to “manual selection.” Statistics were calculated using two-way ANOVA with Holm-Sidak multiple comparisons correction: *, p<0.05; **, p<0.01; ***, p<0.001.

There are a number of ways to select desirable day 17 neuroepithelial rosette structures for further differentiation. We next sought to compare different NPC isolation methods at day 17 of differentiation (Fig. 5A). First, manual neural rosette selection was compared to enzymatic neural rosette selection. Manual rosette selection involved manually scraping away the large, clear cells (neural crest morphology) that surround neural rosette structures to remove these contaminating cell types. For enzymatic rosette selection, the StemCell Technologies STEMdiff Neural Rosette Selection Reagent was used to selectively detach neural rosettes from the dish (Fig. 5B). Immunostaining at day 18 (one day after selection) shows that both manual and enzymatic rosette selection enrich for Pax6+ (Fig. 5C, top row), Nestin+ (Fig. 5C, top and bottom rows) and Oct4− (Fig. 5C, middle row) cells, compared to cells that were not subjected to any NPC selection. Manual selection resulted in fewer Oct4+ cells than rosette selection (Fig. 5C, middle row). Sox2 expression was similar between the three conditions, but there were several Sox2+/Nestin− cells without NPC selection, and a few Sox2+/Nestin− cells after rosette selection (Fig. 5C, bottom row, asterisks). Immunostaining of differentiated neurons at day 40 (after enzymatic rosette selection) shows that 85% (±5.1 SEM) are MAP2+, similar to the 93% MAP2+ neurons resulting from manual selection.

We hypothesized that employing a cell-sorting technique would help decrease non-neuronal cell contamination in our cultures. To test this, we sorted day 17 cells using magnetic affinity cell sort (MACS) technology with a PSA-NCAM antibody. Manually selected NPCs and PSA-NCAM+ cells were plated on Matrigel in neural differentiation media for 23 days and immunostained for various neuronal markers (Fig. 5D). Both conditions (manual selection and PSA-NCAM+) expressed neuronal markers TuJ1, MAP2 and Tau. However, sorted cells (Fig. 5D, right column) had high background levels of non-neuronal cells, indicated by non-neuronal morphology and absence of neuronal markers. Additionally, Tbr1 immunoreactivity was less abundant in MACS preparations compared to manually selected cells (Fig. 5D, middle row). Quantification following PSA-NCAM sorting from these experiments showed 47% (±2.3 SEM) MAP2+ cells.

Because MACS did not improve neuronal purity above other NPC selection strategies, we then tested the ability of FACS to enrich for NPCs by isolating CD184+/CD44−/CD271−/CD24+ cells using the BD Stemflow Human Neural Cell Sorting Kit (based largely on [22]), wherein day 17 cells are dissociated and labeled with these antibodies that mark specific cell populations. CD184+/CD44−/CD271−/CD24+ cells (“NPCs”) and flow-through cells (“non-NPCs”) were maintained in neural progenitor media for 20 days after sorting, followed by immunostaining for Nestin and MAP2 (Fig. 5E). This media, consisting of a DMEM/F12 base with B27, FGF2, EGF, and heparin, supports culture of adherent neural progenitor cells [23]. Fig. 5E shows that FACS reduced the number of Nestin−/MAP2− cells present (asterisks), but was highly stringent and also excluded some cells expressing neuronal markers (arrowheads).

Finally, we compared the gene expression profiles of manual-, rosette-, and FACS-isolated NPCs at day 17 by NanoString (Fig. 5F, G). Gene expression analyses show that enzymatic rosette selection appeared to be most permissive to other cell types, with decreased expression of NPC markers *FoxG1*, *HES1*, *Pax6*, *Vimentin (VIM)*, and *MAP2* (Fig. 5F), and higher expression of non-NPC cell fate markers, including the endodermal marker *AFP* (Fig. 5G). There was also a trend for increased expression of pluripotent cell markers *NANOG* and *Oct4* (*POU5F1*), but this did not achieve significance. FACS-isolated NPCs showed similar overall gene expression to manually isolated NPCs with a few differences, including increased *HES1* and decreased *Tbr2* expression (Fig. 5F). Overall, these three NPC isolation methods each enrich for neural progenitors, with slight differences in NPC purity and identity.

### Consequences of Neural Progenitor Expansion on Neuronal Identity

**Figure 6:**
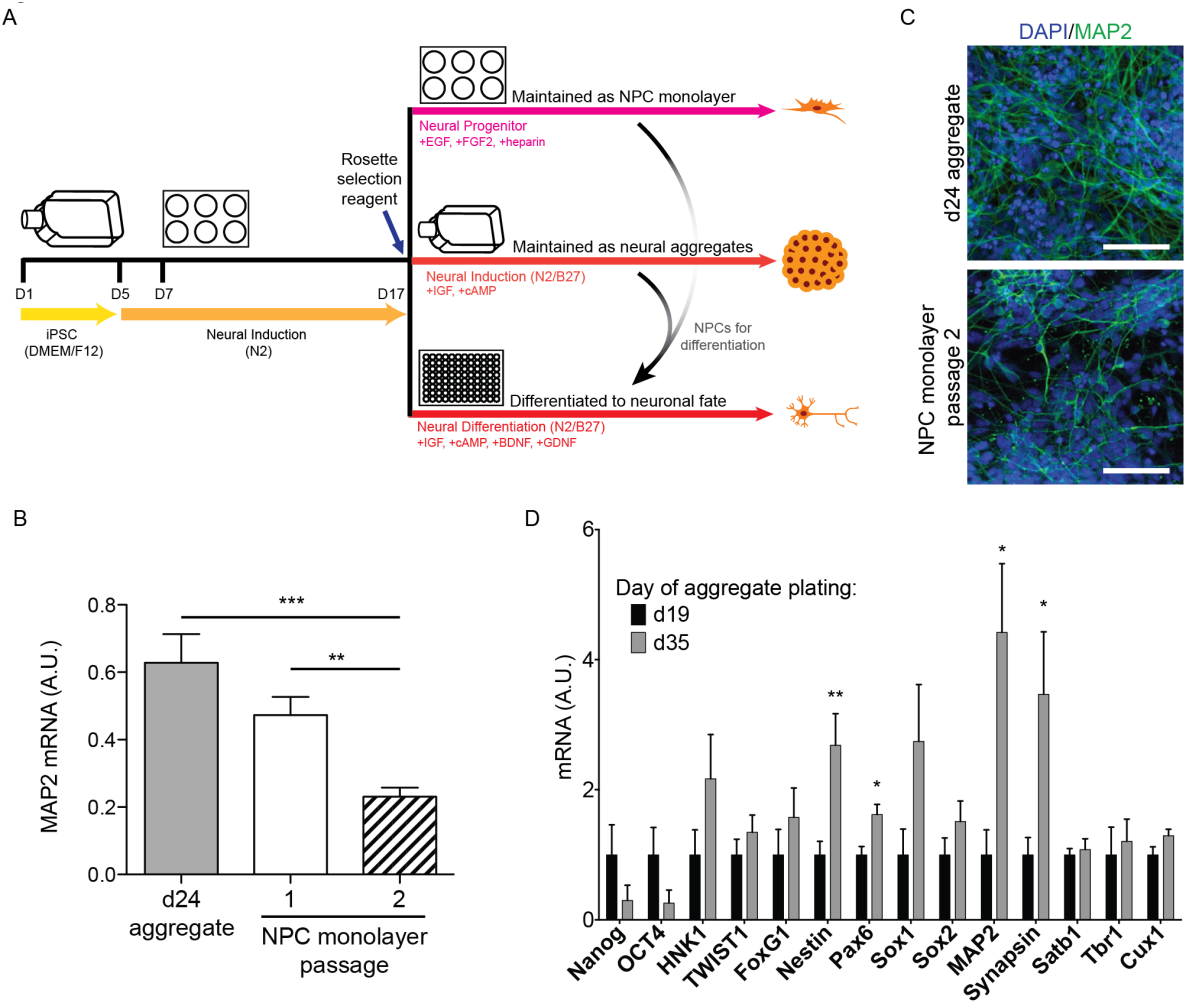
Effects of Neural Progenitor Cell Maintenance and Expansion on Differentiation Efficiency. A) Schematic indicating the time course of differentiation and the techniques used to maintain/differentiate neural progenitor cells (NPCs) after NPC isolation with neural rosette selection reagent at day 17. B) qPCR analysis of MAP2 expression after 16 days of differentiation of day 24 aggregates or passage 1 or 2 monolayer NPCs. Data normalized to *GAPDH*. Data are represented as mean ± SEM, n = 11–20. C) Immunostaining of day 40 (D40) neurons, following differentiation from either day 24 aggregates or passage 2 NPCs. Scale bars = 100 µm. Representative images are shown. D) NanoString analysis of cell fate markers of neural aggregates plated at day 19 or 35, after 16 days of plating in neural differentiation media, normalized to the geometric mean of seven housekeeping genes. Data are represented as mean ± SEM, n = 6. For B and D, significance was determined by student's t-test: *, p<0.05; **, p<0.01; ***, p<0.001.

Differentiation protocols are time-consuming and costly; thus, we hoped to establish a protocol in which we could generate neuronal cells from an expandable NPC pool. This would allow for neuronal differentiation without differentiating cells for 17 days prior to the NPC stage, and for expansion of neural progenitors for increased neuronal yield. Fig. 6A shows the differentiation schematic that was used to culture NPCs. Differentiation was performed using the embryoid aggregate protocol (Fig. 1A) until day 17. At day 17, neural rosettes were selected and isolated using the Neural Rosette Selection Reagent from StemCell Technologies. Harvested cells were either maintained in suspension as neural aggregates in N2/B27 neural induction media, plated for expandable monolayer culture in neural progenitor media, or plated on Matrigel in 96-well plates for final differentiation in neural differentiation media.

We first compared neurons resulting from aggregates to neurons differentiated from monolayer NPCs. Rosette-selected NPCs were maintained on POL-coated plates in neural progenitor media with EGF, FGF2, and heparin. Cells were plated for final neuronal differentiation from a pool of monolayer-maintained NPCs after the first or second passage (∼3–5 days per passage) or directly from day 24 aggregates without passaging. Cells were subsequently maintained in neural differentiation media for 16 days (Fig. 6A). Differentiation of monolayer-maintained NPCs from two subsequent passages showed a trend for decreased *MAP2* mRNA expression after the first passage and significantly lower *MAP2* expression after the second passage compared to neurons derived directly from day 24 aggregates (Fig. 6B). These data indicate decreased potential for neuronal identity with extended monolayer NPC expansion, which could result from expansion of contaminating adherent non-neuronal cells. We also observed a corresponding decrease in MAP2 immunostaining in day 40 neurons derived from NPC monolayer passage 2 compared to neurons derived from day 24 aggregates (Fig. 6C). We then examined effects of suspension neural aggregate progenitor expansion on resulting neuronal identity. NPCs were maintained in suspension as neural aggregates for 2 days after selection (day 19) or 18 days after selection (day 35) before plating for final neuronal differentiation. These cells were cultured in neural differentiation media for 16 days before analysis of mRNA expression by NanoString (Fig. 6D). Prolonged neural aggregate culture did not appreciably alter resulting neuronal identity, as demonstrated by comparable expression of cortical markers *Satb1*, *Tbr1*, and *Cux1* (Fig. 6D). However, the NPC/neural purity of the cultures appeared to be improved with longer neural aggregate culture, shown by higher *nestin (Nes), Pax6, MAP2, and synapsin (SYN)* expression, as well as a trend for lower *NANOG* and *Oct4* (*POU5F1*) expression (Fig. 6D). Thus, if long-term culture and/or expansion of NPCs is desired, maintenance in aggregates may be superior to maintenance as a monolayer.

### The Emergence of Endogenous Astrocytes

**Figure 7:**
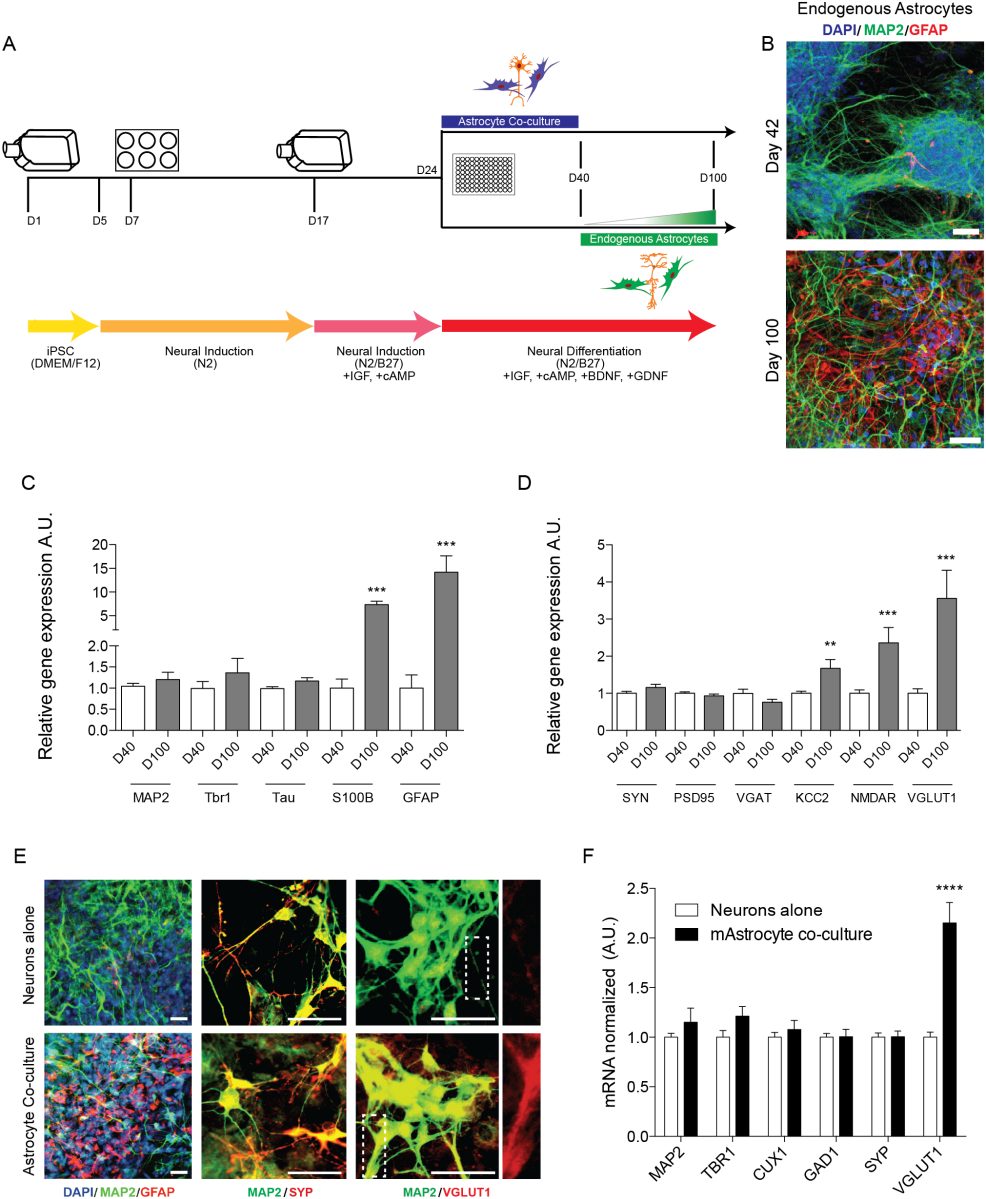
Astrocyte Co-culture Increases Neuronal Maturation and Endogenous Astrocytes Arise at Later Time-Points in Differentiation. A) Schematic of differentiation up to 100 days. For astrocyte co-culture, astrocytes were added to neuronal cultures at ∼day 24 of differentiation. Endogenous astrocytes gradually emerged over the course of 100 days, after day 40. B) Day 42 and day 100 neuronal cultures were immunostained and imaged for GFAP. Scale bars = 50 µm. C) After 40–50 (D40) or 100 days (D100), cells were lysed, RNA extracted, and expression of 150 genes analyzed using the NanoString platform. A subset of neuronal markers (C) and synaptic markers (D) are shown. Data are from at least 6 independent differentiations (3 lines). For day 40–50 n = 29–38, for day 100 n = 15–19. E) Neuron cultures with or without astrocytes were immunostained and imaged using confocal microscopy at day 40. Insets in right column show VGLUT1 staining along the length of a neuronal process. Representative images are shown. Scale bars = 50 µm. F) qPCR was performed using RNA harvested from day 40 cultures. Data normalized to *GAPDH* expression. For neurons alone n = 20 for MAP2, TBR1, CUX1, GAD1, n = 37 for SYP, n = 38 for VGLUT1; for astrocyte co-culture n = 17 for GAD1, n = 18 for MAP2, TBR1, CUX1, n = 27 for SYP, n = 25 for VGLUT1. For C–F, data are represented as mean ± SEM. Significance determined by student’s t-test: **, p<0.01; ***, p<0.001; ****, p<0.0001.

Neuronal markers change over the course of differentiation, such that over time there is an upregulation of synaptic markers. We also were interested in whether endogenous astrocytes emerged in our cultures within 100 days of differentiation (Fig. 7A, green arm). Immunostaining and confocal microscopy of day 42 and day 100 neuronal cultures showed an increase in expression of glial fibrillary acidic protein (GFAP), a marker of astrocytes, at day 100 (Fig. 7B). Additionally, using the NanoString platform, we evaluated RNA harvested from either day 40 or day 100 neuronal cultures for a subset of neuron and astrocyte markers (Fig. 7C) and synaptic markers (Fig. 7D). Day 100 cultures showed a significant increase in expression of the astrocytic markers *GFAP* and *S100B* (Fig. 7C), as well as in markers of mature neurons, *VGLUT1* (*SLC17A7*), *NMDAR* and *KCC2* (Fig. 7D). There was no significant difference in *MAP2*, *Tbr1, Tau*, *SYN, PSD95, or VGAT* between day 40 and day 100 cultures.

### Neuronal Maturation with Astrocyte Co-culture

Lastly, we aimed to address the possible benefits of astrocyte co-culture on differentiation, i.e. if we could accelerate maturation of neuronal cultures before day 100. Differentiated neurons were cultured alone or co-cultured with mouse astrocytes (Sciencell) after plating at day 24 (Fig. 7A, purple arm). Samples were harvested at day 40, before the emergence of endogenous astrocytes. By immunostaining, there were no qualitative differences in MAP2 or SYP expression between culture conditions (Fig. 7E). However, we were able to visualize protein expression of VGLUT1 at day 40 only when differentiated neurons were co-cultured with exogenous astrocytes (Fig. 7E, bottom row). qPCR analysis showed no changes in *MAP2*, *Tbr1, CUX1*, *GAD1* or *SYP* expression, but significantly increased *VGLUT1* (*SLC17A7*) expression at day 40 with astrocyte co-culture (Fig. 7F).

## Discussion

With advancements in iPSC neuronal differentiation, it has been possible to examine human neural development and consequences of neurological disease-associated mutations at the cellular level. However, there exist a multitude of techniques to get from point A (iPSCs) to point B (differentiated neurons). Here, we evaluated several methods that are regularly used to generate forebrain cortical neurons, the “default” neuronal fate generated in the absence of exogenously provided patterning factors. We compared the outcomes of these protocols using gene expression, cell morphology, and protein expression by immunostaining (Table 1). Notably, these protocols resulted in robust expression of forebrain cortical transcription factors with negligible expression of midbrain and hindbrain transcription factors, as assessed by NanoString counts (Figure S1).

**Table 1:** Key comparisons of methods tested.

Optimization Parameter	Key Benchmarks	Results	Notes
*Differentiation Protocols*			
Embryoid Aggregatevs. Monolayer DualSMAD Inhibition	MAP2%, qPCR of neuronal markers	Consistent neuronal yield with over 90% MAP2+ neurons using embryoid aggregate protocol. Decreased MAP2% with dual SMAD inhibition method.	Zeng et al., 2010 **[19]**; Chambers et al., 2009 **[8]**
*Aggregate Formation*			
Manual vs. AggreWell	MAP2%, qPCR ofneuronal markers,brightfield microscopy	Manual formation more variable in aggregate size than Aggrewell. More consistent aggregate size and decreased MAP2% using Aggrewell	Zeng et al., 2010 **[19]**; StemCell Technologies
*Plating Substrates*			
Matrigel vs. POL	MAP2%, qPCR ofneuronal markers,immunostaining ofNPC and iPSC markers	Matrigel promotes aggregate adherence better than POL at D7. Matrigel generates higher percentage of cortical neurons than POL at D40.	Matrigel from BD Biosciences. Lot-to-lot variations in protein content may affect outcome.
*Progenitor Selection*			
Manual/CellSort/RosetteSelection	MAP2%, immunostaining of neuronal markers,Nanostring of NPCand non-neuronalmarkers	Rosette Selection is rapid and efficient but most permissive to non-neural cells. FACS and manual selection are equally effective for eliminating non-neural cells, but FACS is more time-consuming, has a lower yield, and selects NPCs with slightly different marker expression.	Manual: Zeng et al., 2010 **[19]**; Hu et al. 2010 **[9]**; FACs: BD Biosciences, Yuan et al., 2011 **[22]**; Rosette Selection: StemCell Technologies
*Co-culture*			
None vs. Astrocytes	qPCR of neuronalmarkers, Nanostringof neuronal andastrocyte markers,immunostaining ofneuronal markers	Astrocyte-free cultures express neuronal markers, but express less VGLUT1 than co-culture with astrocytes or cultures containing endogenous astrocytes	

The percentage of cells expressing MAP2 was quantified following immunostaining from many of the various differentiation schemes employed here. The manual embryoid aggregate method with manual rosette selection as well as enzymatic rosette selection, generated the highest percentage of MAP2+ cells at day 40 of differentiation (∼93% and ∼85%, respectively). Other differentiation methods resulted in significantly fewer MAP2+ cells, such as monolayer dual SMAD inhibition differentiation (∼45%), AggreWell embryoid aggregate differentiation (∼46%), and PSA-NCAM sorting (∼47%). It has been reported that different iPSC lines can vary in their ability to differentiate into neural cells [9], [24]–[31]. For the embryoid aggregate-based differentiation variations examined herein, we did not observe obvious differences in efficiency of final neuronal differentiation or cell isolation method across cell lines. As this protocol includes steps to minimize or exclude undesirable cell types (e.g. selection of NPCs at day 17 and subsequent NPC culture in suspension), differences in differentiation capacity of different lines are minimized. However, we did note that certain lines differentiated better than others using the dual-SMAD inhibition protocol, with the most promising neuronal differentiations shown here (Table 2).

**Table 2:** Number of iPSC lines, differentiations and well numbers contributing to each figure.

Figure	Lines used	Independentdifferentiations	n
Figure 1	YZ1, TZ1,YK26, fAD 2a	1D: 6	1D: iPS n = 14–15, D17 n = 23–25, D40–50 n = 19–26
Figure 2	YZ1, YK26	*2D: 2	*2D: iPS n = 2–3, D1 n = 3–4, D7 n = 3–4, D11 n = 5–6, D40 n = 5
Figure 3	YZ1, YK26	3B: 4, 3D: 2	3B: manual n = 43, AG3000 n = 21, AG8000 n = 26; 3D: Manual/Aggrewell n = 10
Figure 4	YZ1, YK26	4H/J: 2	4H: Matrigel and POL n = 10; 4J: Matrigel and POL n = 22
Figure 5	YZ1, YK26	5F, G: manual:5; rosette: 18;FACS: 6	5F, G: Manual n = 15; Rosette n = 30; FACS n = 6
Figure 6	YZ1, YK26,fAD 2a, b	6B/D: 2	6B: n = 11–26; 6D: n = 6, both time-points
Figure 7	YZ1, YK26	7C,D: 6; 7F: 5	7C,D: D40–50 n = 29–38, D100 n = 15–19; 7F: Neurons alone n = 20 for MAP2, TBR1, CUX1, GAD1, n = 37 for SYP, n = 38 for VGLUT1; Astrocyte Co-culture n = 17 for GAD1, n = 18 for MAP2, TBR1, CUX1, n = 27 for SYP, n = 25 for VGLUT1

*10/10 differentiations without dissociation failed. 3/5 differentiations with dissociation yielded MAP2+ cells.

At both plating steps of the embryoid aggregate protocol, Matrigel appears to be a superior substrate for promoting cell adherence and acquisition of neuronal identity. Use of Matrigel in the second plating generates >90% MAP2+ cells at day 40 of differentiation, compared to 56% MAP2+ cells using POL for the second plating. Plating cells on Matrigel at the second step leads to higher *MAP2* mRNA expression compared to the POL plating, despite whether the cells are plated as aggregates or dissociated and plated as a monolayer. Matrigel likely serves as a better substrate due to its complex composition, which includes laminin, collagen IV and entactin, as well as a variety of growth factors that may promote neuronal differentiation. Notably, we have found that the lot-to-lot variability in Matrigel protein concentration is important for its differentiation- and adherence-promoting capability in the second plating step. Matrigel lots with higher initial protein concentrations are often more suitable for neuronal differentiation, even when plated at the same absolute protein levels.

Isolation of NPCs may be done by a variety of methods, four of which we compared here: manual selection, enzymatic rosette selection, PSA-NCAM MACS, and FACS. PSA-NCAM sorting was less effective than manual NPC selection, with decreased neuronal purity in PSA-NCAM+ sorted populations. Each of the remaining methods serves to enrich for NPCs, with some differences. Rosette selection appears to be more permissive to undesirable cell types, including pluripotent cells and endodermal cells, than manual selection or FACS. However, this method still generates neurons with high purity. The increased *HES1* expression with FACS could reflect increased purity of proliferative, undifferentiated neuroepithelial cells with FACS isolation [32], [33]. Decreased *Tbr2* expression with FACS isolation suggests that this method may enrich for earlier neural progenitors at the expense of decreased enrichment for intermediate progenitors [34].

The less selective nature of the neural rosette selection reagent should be balanced with the cost (in both time and money) of each method. The manual selection method can be the most time-consuming and requires an experienced user, but requires no additional reagents and thus has the lowest reagent cost. The rosette selection method is fastest, but requires use of a proprietary reagent and so comes with a moderate cost. Finally, FACS selection is somewhat time-consuming and is the most expensive method. FACS also greatly reduces the total yield of viable cells, as there is significant cell loss due to prolonged dissociation and sorting time. The decision to choose one of these methods should be determined by the experimental setup and subsequent use of the isolated NPCs. If a highly sensitive and/or expensive method will be used on the isolated NPCs and purity is of the utmost importance, FACS isolation may be optimal. For experiments with few cell lines, manual selection is the cheapest and most effective method. If many different hiPSC lines are being differentiated, manual rosette selection would be time-prohibitive and rosette selection may be a better option. For an experiment in which NPCs will be further purified as neural aggregates (where many non-NPC fates will adhere while NPCs float as aggregates), rosette selection should be acceptable.

Culturing cells as NPCs has the advantage of expanding the neural progenitor pool for subsequent neuronal differentiation without having to repeat days 1–17 of differentiation. This can save time and resources while increasing neuronal yield per differentiation experiment. If expansion of cells at the NPC stage is desired, our data suggest that maintenance as neural aggregates is superior to maintenance in a monolayer. Differentiation of later passage cells from aggregates show increased purity without obvious alterations in neuronal identity (as assayed by our 150-probe NanoString profile), whereas extended culture in monolayer decreased neuronal identity of the resulting cells. This may be related to the suspension nature of aggregate culture, wherein many contaminating cell types (e.g. neural crest cells) will preferentially adhere to the flask, whereas desirable cell types (e.g. NPCs) will be maintained as suspended aggregates. There also is a possibility that the slightly different media formulations of N2/B27 neural induction media versus neural progenitor media may alter cell fate and differentiation capacity (e.g. N2, cAMP, IGF-1 only in the former and FGF2, EGF only in the latter). Of note, neural aggregate size increases as cells proliferate, which eventually limits nutrient access for cells inside the aggregate. To allow cells to continue proliferating while maximizing access of cells to nutrients, neural aggregates can be broken up by gentle trituration [12]. Other groups have reported the establishment of NPC lines from hESCs or hiPSCs, often using more than one purification strategy to generate a highly pure and homogenous NPC population [23], [35]–[41]. These strategies could be considered to generate NPC lines for longer-term use, but it is less sustainable to execute multiple purification techniques for many lines over multiple differentiations.

We also sought to study the emergence of endogenous astrocytes from neuronal cultures and examine the effect of exogenous astrocytes on iPSC-derived neurons. Over differentiation time, remaining neural progenitor cells begin to produce astrocytes, shown by increasing astrocyte marker expression and immunostaining by day 100. This confirms data presented by other groups showing emergence of astrocytes with long-term differentiation of hiPSC-derived neural progenitors [42]–[44]. Concurrently, there is an increase in the excitatory neuronal marker *VGLUT1* (*SLC17A7*) (but not *SYN*) expression. Co-culture of human iPSC-derived neurons with mouse astrocytes promoted increased expression of *VGLUT1* (*SLC17A7*) at day 40, without affecting other neuronal subtype and synaptic markers. This suggests that exogenous astrocyte co-culture promotes the maturation of iPS-derived neurons without altering cell fate. These data are consistent with reports of accelerated hESC- and hiPSC-derived neuronal maturity with astrocyte co-culture [45], [46]. Addition of exogenous astrocytes has the advantage of accelerating neuronal maturation, which may be desirable depending on the phenotype to be studied.

Achieving a “standard” protocol for neural differentiation across laboratories is unrealistic, due to the wide range of studied phenotypes and continual development of new protocols. Because small variations in methods can have dramatic effects on the resulting cells (and studied cellular properties), it is imperative that groups utilizing iPSC-derived neural cells carefully report how differentiation was performed and thoroughly characterize the resulting cell populations. The data included here provide a framework upon which researchers can base decisions regarding differentiation protocols. We hope this may aid in selection of optimal protocols, promote awareness of the many variables that can affect differentiation, and encourage detailed reporting of differentiation methods in published studies.

### Experimental Procedures

#### iPSC Reprogramming and Generation

Human iPSCs were obtained from the UCONN Stem Cell Core. Lines YK26, YZ1 and TZ1 were generated by retroviral vectors containing the reprogramming factors OCT4, SOX2, NANOG and LIN28 or c-Myc and KLF4 [19]. Additional lines were reprogrammed by retroviral vectors containing the reprogramming factors OCT4, SOX2, c-Myc and KLF4 in conjunction with the Harvard Stem Cell Institute as previously described [21].

#### iPSC Karyotype Analysis and Characterization

The NanoString nCounter Human Karyotype Panel CNV CodeSet was used to assay iPSC genomic DNA every ∼10 passages in order to ensure a stable chromosome number over time.

#### iPS Cell Culture

iPSCs were cultured in iPSC media as previously described [21]. FGF2 (Millipore) was added fresh daily at 10 ng/ml. Cells were maintained at 37°C/5% CO_2_ and were split as necessary based on colony growth (∼6 days). iPSCs were manually groomed by removing any colonies with irregular borders, spontaneous differentiation or transparent centers, prior to splitting. iPSCs were maintained on a mouse embryonic fibroblast (MEF) feeder layer at 1.7–2.0×10^5^ cells/well of a 6-well plate (Globalstem).

#### Embryoid Aggregate Differentiation Protocol

For the induction of forebrain neurons, iPSCs were differentiated using an embryoid body-based protocol [19], further optimized here. Colonies with irregular borders, spontaneous differentiation or transparent centers were removed prior to splitting. iPSC colonies were dissociated from MEFs at day 1 with collagenase (StemCell Technologies) and cultured as aggregates for 4 days in suspension with iPSC media (no FGF2), with media changes every day. At day 5, aggregates were washed 1X with N2 Neural Induction media and then fed with N2 Neural Induction media. On day 7, aggregates were plated on either Matrigel-coated culture dishes (used per the manufacturer’s instructions, BD Biosciences) or poly-ornithine (4 µg/cm^2^) and laminin-coated plates (1 µg/cm^2^), at about 20–30 aggregates/well. Cells were fed every 2 days with N2 Neural Induction media. Over the course of 10 days, primitive neuroepithelial (NE) structures were formed. By day 17 definitive NE structures were present and rosettes selected.

#### Neural Rosette Selection

Neural rosettes were selected manually, selected with STEMDiff Neural Rosette Selection reagent (used per the manufacturer’s instructions, StemCell Technologies), or purified by MACS/FACS (further information below). For manual selection, cells with non-rosette morphology were scratched off culture plates using either sterile glass pipettes or sterile plastic pipette tips, followed by aspiration of undesirable material. Remaining rosettes were then scraped from the plate for further use. For each selection method, neural progenitor cells (NPCs) were either dissociated and plated for further differentiation or re-cultured in non-adherent culture flasks. Alternatively, NPCs were cultured as an adherent monolayer.

#### Neural Progenitor Cell Monolayer Culture

NPCs were maintained in neural progenitor media (+FGF2, EGF, heparin) and passaged 1∶3 every 3–5 days or as necessary when confluent. Cells were split onto poly-ornithine (4 µg/cm^2^) and laminin (1 µg/cm^2^) coated plates. Plates were coated overnight in a humidified 37°C incubator.

#### Neural Aggregate Culture

After NPC selection, cells were cultured in suspension and fed with N2/B27 neural induction media with cAMP and IGF-1. At day 24 (or as otherwise noted) cells maintained as aggregates were either plated as aggregates (3–5 aggregates/well of 96 well plate) or dissociated to single cells with accutase (Invitrogen) (40,000–50,000 cells/well of 96 well plate) and plated on Matrigel for final differentiation in Neural Differentiation media with ROCK inhibitor (Stem RD, 10 µM). Cells plated at day 17 were also switched to neural differentiation media at day 24 for the remainder of the experiment. A full media change was performed every 2–3 days.

#### Astrocyte Co-culture

Mouse astrocytes (Sciencell) were plated on top of differentiated human neurons at day 26 in a 1∶1 media mix of Neural Differentiation media and Astrocyte media (Sciencell). Approximately 120,000 cells/cm^2^ were plated. Prior to plating, mouse astrocytes were maintained per the manufacturer’s directions.

#### Monolayer Differentiation Protocol

Using an alternate method for the induction of forebrain neurons, iPSCs were differentiated using a monolayer protocol [8], [10]. iPSCs were manually groomed by removing any colonies with irregular borders, spontaneous differentiation or transparent centers. To initiate differentiation, cells were dissociated with accutase (Invitrogen) for 30 minutes at room temperature. The cells were then triturated to form a single cell suspension and subsequently filtered through a 0.45 µm cell strainer to remove any cell clumps. Remaining cells on the plate were rinsed with additional iPSC media. Cells were washed and centrifuged (200 g, 5 minutes) 2x and then resuspended in 10 mL iPSC media with ROCK inhibitor (StemRD, 10 µM). The cell suspension was then plated on a pre-coated gelatin 10 cm plate, with a density of less than 200,000 cells/cm^2^. 10 cm dishes were then incubated at 37°C for 30 minutes to allow MEFS time to adhere to the gelatin, without substantial adherence of iPSCs. After 30 minutes, suspended cells were washed with iPSC media + 10 µM ROCK inhibitor and centrifuged (200 g, 5 minutes). Collected cells were resuspended with MEF conditioned media + 10 µM ROCK inhibitor. Cells were re-plated as a monolayer with a concentration of 20,000 cells/cm^2^ in MEF conditioned media, supplemented with FGF2 (10 ng/mL). After cells reached 90% confluency, media was changed to 3N neural induction media (defined below) supplemented with Noggin (200 ng/mL) and SB431542 (10 µM) [10]. Cells were split at day 11 using dispase and re-plated in neural differentiation media onto 96-well plates coated with Matrigel.

#### Aggregate formation using AggreWell

Aggregates were formed using either 400 or 800 µm well plates. Plates were used per the manufacturer’s instructions to form aggregates of either 3,000 or 8,000 cells/aggregate. 24 hours after AggreWell plating (day 2), aggregates were resuspended in low-adherence flasks and cultured in the appropriate medias listed above.

### Medias

#### MEF Medium

435 mL DMEM (Invitrogen), 5 mL 100x Penicillin/Streptomycin (Invitrogen), 5 mL 100x L-glutamine (Invitrogen), 50 mL FBS (Invitrogen).

#### iPS Medium

390 mL DMEM/F12, 100 mL KOSR (Invitrogen), 5 mL 100x Penicillin/Streptomycin/Glutamine (Invitrogen), 5 mL 100x MEM-NEAA (Invitrogen), 50 µM b-mercaptoethanol (Invitrogen), with the addition of fresh FGF2 (Millipore, 10 ng/mL) to the medium.

#### N2 Neural Induction Medium

490 mL DMEM/F12 (Invitrogen), 5 mL N2 supplement (Invitrogen), 5 mL 100x MEM-NEAA (Invitrogen), and Heparin (Sigma-Aldrich, 2 µg/mL).

#### N2/B27 Neural Induction Medium

480 mL DMEM/F12, 5 mL N2 supplement (Invitrogen), 10 mL B27 supplement (Invitrogen), 5 mL MEM-NEAA (Gibco) and 2 µg/ml Heparin (Sigma-Aldrich), with the addition of fresh cAMP (1 µM) (Sigma) and IGF1 (PeproTech, 10 ng/mL) to the medium.

#### Neural Differentiation Medium

490 mL Neurobasal medium (Invitrogen), 5 mL N2 supplement (Invitrogen), 5 mL 100x MEM-NEAA (Invitrogen), and 10 mL B27 supplement (Invitrogen), with the addition of fresh cAMP (Sigma, 1 µM), BDNF, GDNF, and IGF-1 (all PeproTech, 10 ng/mL) to the medium.

#### Neural Progenitor Medium

350 mL DMEM (Invitrogen), 150 mL F12 (Invitrogen), 5 mL 100x sodium pyruvate (Invitrogen, only if not included in DMEM formulation), 5 mL 100x Penicillin/Streptomycin/Glutamine (Invitrogen), 10 mL B27 supplement (Invitrogen) with the addition of fresh EGF (Sigma, 20 ng/mL), FGF2 (Millipore, 20 ng/ml), and heparin (Sigma, 5 µg/ml) to the medium.

#### MEF Conditioned Medium

2.8×10∧6 mouse embryonic fibroblasts (GlobalStem) were plated on a gelatin-coated dish (1 hour at room temperature) in MEF media. 24 hours later, cells were washed 1X with iPS media and fed with fresh iPS media. Media was incubated for 24 hours and then collected. Additional iPS media was conditioned every 24 hours for up to 2 weeks. All media was pooled and sterile-filtered before use. 10 ng/mL of FGF2 was added fresh before use.

#### 3N Neural Induction Medium

485 mL DMEM/F12, 5 ml 100x MEM-NEAA (5 μg/mL), 5 mL N2 supplement (Invitrogen), 10 mL B27 supplement (Invitrogen), insulin (Sigma, 5 µg/mL), 50 µM b-mercaptoethanol (5 µg/mL), 5 mL 100x Penicillin/Streptomycin/Glutamine (5 µg/mL).

### qPCR

RNA was purified from individual samples and processed through a PureLink RNA Mini Kit (Ambion), followed by reverse transcription using SuperScript II (Invitrogen). qPCR was performed using Fast SYBR Green Master Mix (Applied Biosystems) and run on a ViiA 7 System (Applied Biosystems). Samples were assayed with 3 technical replicates. Data was analyzed using the ΔΔ*C*_T_ method and expression was normalized to GAPDH expression [47]. Primer efficiency was calculated for each pair of primers and the slope of the dilution line was found to be within the appropriate range. Dissociation curves also showed single peak traces, indicating template-specific products.

### Primers

*Oct4*- Forward: TGGGCTCGAGAAGGATGTG; Reverse: GCATAGTCGCTGCTTGATCG

*MAP2*- Forward: AACCGAGGAAGCATTGATTG; Reverse: TTCGTTGTGTCGTGTTCTCA

*Tbr1*- Forward: TCACCGCCTACCAGAACAC; Reverse: GTCCATGTCACAGCCGGT

*GAPDH*- Forward: GGGAGCCAAAAGGGTCATCA; Reverse: TGGTTCACACCCATGACGAA

*CUX1*- Forward: GATGCCACCGCAACGGTAT; Reverse: GGACTGCTCACTTTCATCCTG

*VGLUT1*- Forward: ACTCAGCTCCAGCGTCTCC; Reverse: GAGTTTCGGAAGCTAGCGG

*GAD1*- Forward: AGGAGAGGCAATCCTCCAAGA; Reverse: ATCCCGGTCGCTGTTTTCAC

*SYP*- Forward: AGGGAACACATGCAAGGAG; Reverse: CTTAAACACGAACCACAGG

### NanoString analysis

We utilized a custom 150 gene probe set designed by NanoString Technologies (nCounter Gene Expression Assay) to analyze gene expression for a large number of genes from an individual sample. All assays were performed following NanoString protocols. The initial hybridization reactions were carried out with 100–1000 ng RNA. Post-hybridization samples were processed using the nCounter Prep-station. Following run completion, the cartridge was scanned at max resolution (<1000 images/sample) using the nCounter Digital Analyzer. Data were analyzed using the nSolver Analysis Software and normalized to a set of 7 house-keeping genes (HK) or to the total gene set, as noted. HK genes: *GAPDH, GUSB, HPRT1, LDHA, POLR2A, RPL13a* and *RPL27*. Probe sequences are listed in Table 3.

**Table 3:** Probe sequences for NanoString assay.

NanoString genes	Probe Sequence
*AFP*	GGAGCGGCTGACATTATTATCGGACACTTATGTATCAGACATGAAATGACTCCAGTAAACCCTGGTGTTGGCCAGTGCTGCACTTCTTCATATGCCAACA
*Cux1*	ACAAACAGCCCTGGAAAAAACTCGAACAGAATTATTTGACCTGAAAACCAAATACGATGAAGAAACTACTGCAAAGGCCGACGAGATTGAAATGATCATG
*EN1*	GCAGCATTTTTGAAAAGGGAGAAAGACTCGGACAGGTGCTATCGAAAAATAAGATCCATTCTCTATTCCCAGTATAAGGGACGAAACTGCGAACTCCTTA
*FoxG1*	CTGACAAGTCTATCTCTAAGAGCCGCCAGATTTCCATGTGTGCAGTATTATAAGTTATCATGGAACTATATGGTGGACGCAGACCTTGAGAACAACCTAA
*GFAP*	AAGCAGATGAAGCCACCCTGGCCCGTCTGGATCTGGAGAGGAAGATTGAGTCGCTGGAGGAGGAGATCCGGTTCTTGAGGAAGATCCACGAGGAGGAGGT
*HES1*	ATCTGAGCACAGAAAGTCATCAAAGCCTATTATGGAGAAAAGACGAAGAGCAAGAATAAATGAAAGTCTGAGCCAGCTGAAAACACTGATTTTGGATGCT
*HES5*	CAGCCTGTAGAGGACTTTCTTCAGGGCCCGTAGCTGCTGGGCGTACCCCTGGCAGGCGGGCTGTGCCGCGGGCACATTTGCCTTTTGTGAAGGCCGAACT
*HNK1*	GAGGGAGGCCTGAGCACACTGCTTTGGAAATTATTCTAAACACAAAAAAGGGAAAGAAAATGTTATTTCTCCCTAAGTCAGGAGCATGCAGAGCTAGCCC
*HB9*	CCTGGGCGCTTCCCTTTTAAGCAAGGGCGCCTCACCTGCTCTTCAAGAAACAGCGAGAGGGAGACCCAGGGGGCTGAAACTTGAACTCTGGTTCTTTTAA
*HOXB6*	CACCCATTCCTTTAAATCCGGAGGGGGAAAAAATCCCAAGGTCTGCAAAGGCGCGGCGCTCGGACTATAAAACACAACAAATCATAAACCCGGCGGAGCA
*HOXB13*	CCACCAGGGTTCCCAAAGAACCTGGCCCAGTCATAATCATTCATCCTGACAGTGGCAATAATCACGATAACCAGTACTAGCTGCCATGATCGTTAGCCTC
*KCC2*	ATGAGAGCGACATCTCAGCTTACACCTATGAGAAGACGTTGGTGATGGAGCAGCGTTCCCAGATCCTCAAACAGATGCATTTAACCAAGAATGAGCGGGA
*MAP2*	TACTCTGTATGCTGGGATTCCGAGGTTCCAACACACTGTTACAAATCTGTGGGGGGTTTCTTTCTTCTGATAATTCTAGAGCCTGTTACCATAGAAAGGC
*MYOD1*	TGTAATCTATTCCTGTAAATAAGAGTTGCTTTGCCAGAGCAGGAGCCCCTGGGGCTGTATTTATCTCTGAGGCATGGTGTGTGGTGCTACAGGGAATTTG
*Nanog*	TGCAGGCAACTCACTTTATCCCAATTTCTTGATACTTTTCCTTCTGGAGGTCCTATTTCTCTAACATCTTCCAGAAAAGTCTTAAAGCTGCCTTAACCTT
*Nestin*	CAGAGAATCACAAATCACTGAGGTCTTTAGAAGAACAGGACCAAGAGACATTGAGAACTCTTGAAAAAGAGACTCAACAGCGACGGAGGTCTCTAGGGGA
*NMDAR*	TTCAAGAGAGTGCTGATGTCTTCCAAGTATGCGGATGGGGTGACTGGTCGCGTGGAGTTCAATGAGGATGGGGACCGGAAGTTCGCCAACTACAGCATCA
*Oct4*	AAGTTCTTCATTCACTAAGGAAGGAATTGGGAACACAAAGGGTGGGGGCAGGGGAGTTTGGGGCAACTGGTTGGAGGGAAGGTGAAGTTCAATGATGCTC
*Pax6*	GGGAATTAAAGGCCTTCAGTCATTGGCAGCTTAAGCCAAACATTCCCAAATCTATGAAGCAGGGCCCATTGTTGGTCAGTTGTTATTTGCAATGAAGCAC
*PSD95*	TGCCCTGAAGAATGCGGGTCAGACGGTCACGATCATCGCTCAGTATAAACCAGAAGAGTACAGCCGATTCGAGGCCAAGATCCACGACCTTCGGGAACAG
*S100B*	AGAAGGCCATGGTGGCCCTCATCGACGTTTTCCACCAATATTCTGGAAGGGAGGGAGACAAGCACAAGCTGAAGAAATCCGAACTCAAGGAGCTCATCAA
*Satb1*	TTCCGAAATCTACCAGTGGGTACGCGATGAACTGAAACGAGCAGGAATCTCCCAGGCGGTATTTGCACGTGTGGCTTTTAACAGAACTCAGGGCTTGCTT
*Sox1*	AAAGCGTTTTCTTTGCTCGAGGGGACAAAAAAGTCAAAACGAGGCGAGAGGCGAAGCCCACTTTTGTATACCGGCCGGCGCGCTCACTTTCCTCCGCGTT
*Sox2*	AAAGCGTTTTCTTTGCTCGAGGGGACAAAAAAGTCAAAACGAGGCGAGAGGCGAAGCCCACTTTTGTATACCGGCCGGCGCGCTCACTTTCCTCCGCGTT
*Synapsin*	GGATCTACTTCTGTTTTAGAACCTCCACATTCCTGAAGACCTCCGCCCCTGGTTTCCCCAGAGGGCGTTTTCCTTCCTGGAAGTGCCCAAATACCAGGCA
*Tau*	ATTGGGTCCCTGGACAATATCACCCACGTCCCTGGCGGAGGAAATAAAAAGATTGAAACCCACAAGCTGACCTTCCGCGAGAACGCCAAAGCCAAGACAG
*Tbr1*	GCCGTCTGCAGCGAATAAGTGCAGGTCTCCGAGCGTGATTTTAACCTTTTTTGCACAGCAGTCTCTGCAATTAGCTCACCGACCTTCAACTTTGCTGTAA
*Tbr2*	TCTCTAGATTCCAATGATTCAGGAGTATACACCAGTGCTTGTAAGCGAAGGCGGCTGTCTCCTAGCAACTCCAGTAATGAAAATTCACCCTCCATAAAGT
*TWIST1*	CAACTCCCAGACACCTCGCGGGCTCTGCAGCACCGGCACCGTTTCCAGGAGGCCTGGCGGGGTGTGCGTCCAGCCGTTGGGCGCTTTCTTTTTGGACCTC
*VGAT*	CAGGCTGGAACGTGACCAACGCCATCCAGGGCATGTTCGTGCTGGGCCTACCCTACGCCATCCTGCACGGCGGCTACCTGGGGTTGTTTCTCATCATCTT
*VGLUT1*	TCGGCTACTCGCACTCCAAGGGCGTGGCCATCTCCTTCCTGGTCCTAGCCGTGGGCTTCAGCGGCTTCGCCATCTCTGGGTTCAACGTGAACCACCTGGA
*Vimentin*	GAGGAGATGCTTCAGAGAGAGGAAGCCGAAAACACCCTGCAATCTTTCAGACAGGATGTTGACAATGCGTCTCTGGCACGTCTTGACCTTGAACGCAAAG
***Housekeeping genes:***	
*B2M*	CGGGCATTCCTGAAGCTGACAGCATTCGGGCCGAGATGTCTCGCTCCGTGGCCTTAGCTGTGCTCGCGCTACTCTCTCTTTCTGGCCTGGAGGCTATCCA
*GAPDH*	TCCTCCTGTTCGACAGTCAGCCGCATCTTCTTTTGCGTCGCCAGCCGAGCCACATCGCTCAGACACCATGGGGAAGGTGAAGGTCGGAGTCAACGGATTT
*GUSB*	CGGTCGTGATGTGGTCTGTGGCCAACGAGCCTGCGTCCCACCTAGAATCTGCTGGCTACTACTTGAAGATGGTGATCGCTCACACCAAATCCTTGGACCC
*HPRT1*	TGTGATGAAGGAGATGGGAGGCCATCACATTGTAGCCCTCTGTGTGCTCAAGGGGGGCTATAAATTCTTTGCTGACCTGCTGGATTACATCAAAGCACTG
*LDHA*	AACTTCCTGGCTCCTTCACTGAACATGCCTAGTCCAACATTTTTTCCCAGTGAGTCACATCCTGGGATCCAGTGTATAAATCCAATATCATGTCTTGTGC
*POLR2A*	TTCCAAGAAGCCAAAGACTCCTTCGCTTACTGTCTTCCTGTTGGGCCAGTCCGCTCGAGATGCTGAGAGAGCCAAGGATATTCTGTGCCGTCTGGAGCAT
*RPL13a*	AGTCCAGGTGCCACAGGCAGCCCTGGGACATAGGAAGCTGGGAGCAAGGAAAGGGTCTTAGTCACTGCCTCCCGAAGTTGCTTGAAAGCACTCGGAGAAT
*RPL27*	GGGCCGGGTGGTTGCTGCCGAAATGGGCAAGTTCATGAAACCTGGGAAGGTGGTGCTTGTCCTGGCTGGACGCTACTCCGGACGCAAAGCTGTCATCGTG

### Antibodies

Immunostaining was performed with the following antibodies: Abcam: [MAP2 (1∶5000), Oct4 (1∶1000), Tbr1 (1∶200), Sox2 (1∶1000), SYP (1∶250), VGLUT1 (1∶500), GFAP (1∶1000)]; Millipore, Tbr2 (1∶500); Dako, Tau (1∶200); Sigma, TuJ1 (1∶1000); R+D, Nestin (1∶1000); Covance, Pax6 (1∶300) and Novus, Sox 1 (1∶200). Secondary antibodies were supplied by Jackson ImmunoResearch: anti-chicken Cy2/Cy3/Cy5, anti-rabbit Cy2/Cy3, anti-mouse Cy2/Cy3. Invitrogen, TOPRO3 & DAPI (nuclear markers, 1∶1000).

### Immunocytochemistry and microscopy

Cultures were fixed with 4% paraformaldehyde, followed by membrane permeabilization and blocking with 0.1% Triton X-100 in donkey serum (Jackson ImmunoResearch). Samples were incubated with primary and secondary antibodies (see Antibodies) overnight and 1 hour, respectively. Imaging was performed using a Zeiss LSM710 confocal microscope and images were acquired using ZEN black software. Software was used to pseudo-color images and add scale bars. Quantified MAP2 immunostaining was performed blind on at least 3 images per condition, with at least 200 cells counted per image, using ImageJ software (NIH).

### MACS

Day 17 embryoid aggregate-differentiated cells were utilized for MACS. Cells were dissociated to single cells using accutase (Invitrogen) +10 µM ROCK inhibitor (StemRD) for 30–45 minutes. Cell clumps were removed using a 70 µm strainer (Pre-separation filter, Miltenyi). Cells were sorted per the manufacturer’s instructions using Anti-PSA-NCAM Microbeads (Miltenyi) and related equipment (MS columns and MACS Separator, Miltenyi).

### FACS

Day 17 embryoid aggregate-differentiated cells were utilized for FACS. Cells were dissociated using accutase (Invitrogen) for 25 minutes and treated per the manufacturer’s protocol (Human Neural Cell Sorting Kit, BD Biosciences). The kit was used to isolate CD184+/CD44−/CD271−/CD24+ neural stem cells, which were separated from neural crest and other non-neuronal cells using a BD FACSAria cell sorter. Cells were either harvested after sorting for RNA analysis or plated on Matrigel for immunostaining and confocal microscopy analysis.

### Statistics

Data was analyzed using GraphPad PRISM 5/6 software. Values are expressed as means ±S.D. or ±SEM, as indicated by figure legend text. Statistical significance was tested by either an unpaired Student's *t*-test (two-tailed), by one-way ANOVA with a Tukey’s post-test, or by two-way ANOVA with Holm-Sidak multiple comparisons correction (as indicated by figure legend text). Statistically significant differences were determined by *P* values less than 0.05.

## Supporting Information

Figure S1 **Expression of forebrain cortical vs. mid- or hindbrain transcription factors.** Day 17 (NPC) or day 40 (neuron) RNA (same samples as used in Figures 5 or 6D, respectively). NanoString counts show robust cortical transcription factor expression (FoxG1, Sox1, Sox2, Tbr1, Tbr2, HES1, HES5) and negligible expression of non-cortical transcription factors (EN-1, HB9, HOXB6, HOXB13).
(TIF)
