# Supplementary figures and images for: Comparison and Optimization of hiPSC Forebrain Cortical Differentiation Protocols
Source: PLoS One. 2014 Aug 28;9(8):e105807. doi: 10.1371/journal.pone.0105807 (PMC4148335; doi:10.1371/journal.pone.0105807)

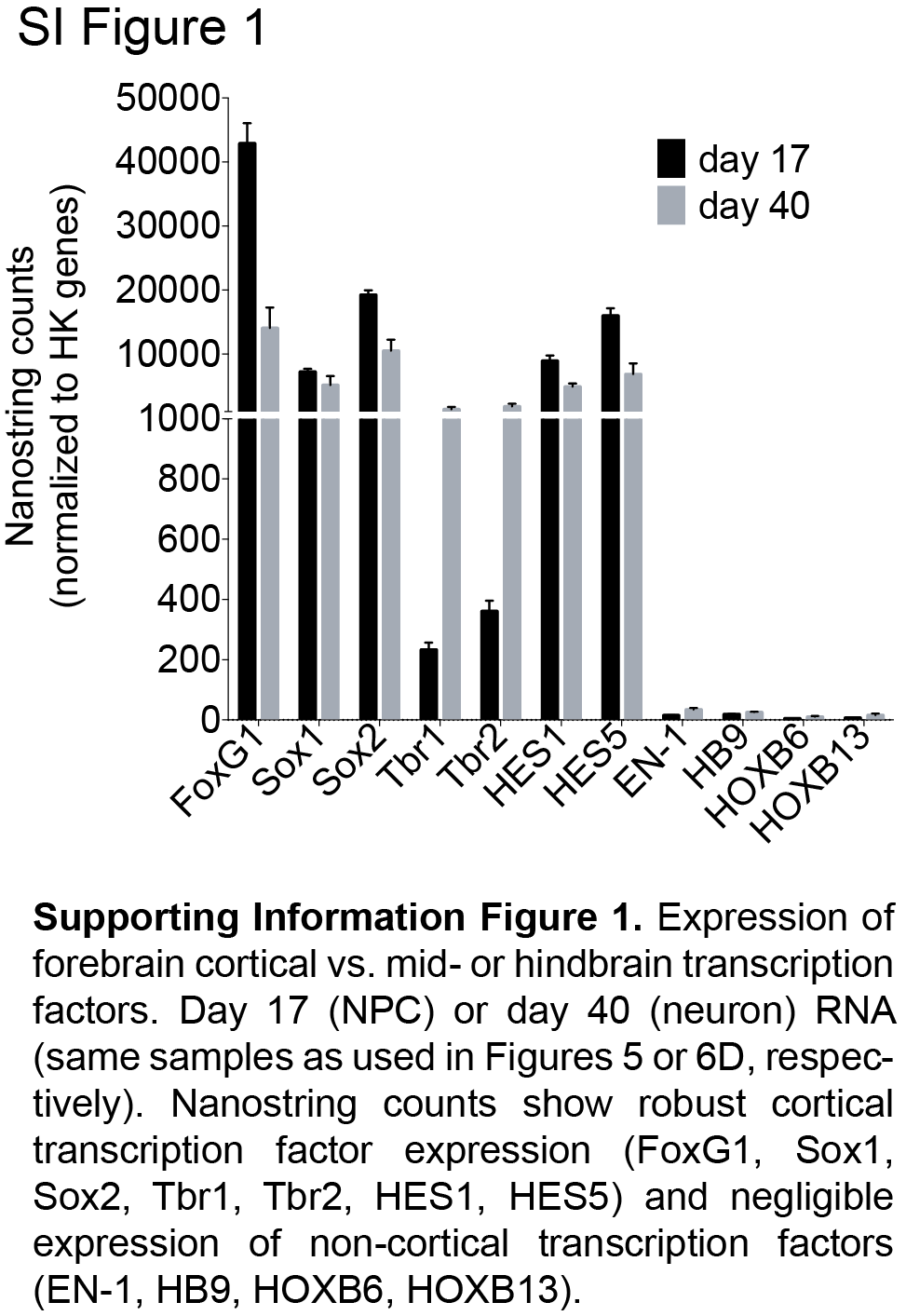

Supplement: Figure S1 — Expression of forebrain cortical vs. mid- or hindbrain transcription factors. Day 17 (NPC) or day 40 (neuron) RNA (same samples as used in Figures 5 or 6D, respectively). NanoString counts show robust cortical transcription factor expression (FoxG1, Sox1, Sox2, Tbr1, Tbr2, HES1, HES5) and negligible expression of non-cortical transcription factors (EN-1, HB9, HOXB6, HOXB13). (TIF) [file pone.0105807.s001.tif]
